# Leveraging network analysis to determine sex differences in factors associated with frailty among older adults living alone

**DOI:** 10.1186/s12877-023-03755-y

**Published:** 2023-01-21

**Authors:** Chiyoung Lee, Yeon-Hwan Park, Belong Cho

**Affiliations:** 1 School of Nursing & Health Studies, University of Washington Bothell, 18115 Campus Way NE, Bothell, WA 98011 USA; 2 College of Nursing, Seoul National University, 103 Daehak-Ro, Jongno-Gu, Seoul, Republic of Korea 03080; 3 The Research Institute of Nursing Science, College of Nursing, Seoul National University, 103 Daehak-Ro, Jongno-Gu, Seoul, Republic of Korea 03080; 4 Department of Family Medicine, College of Medicine, Seoul National University, 103 Daehak-Ro, Jongno-Gu, Seoul, Republic of Korea 03080; 5 Health Promotion Center, Seoul National University Hospital, 101 Daehak-Ro, Jongno-Gu, Seoul, Republic of Korea 03080

**Keywords:** Epidemiology, Frailty, Network analysis, Korea

## Abstract

**Background:**

Frailty is a complex geriatric syndrome typically characterized by multiple underlying etiological factors. We determined the contributing factors, by sex, using a network analysis.

**Methods:**

The study sample consisted of a cross-sectional cohort of community-dwelling older adults aged ≥ 65 years living alone in a Korean city (*N* = 1,037). Frailty was assessed via the Korean Frailty Index. Participants were assessed for sociodemographic, health-related, mental and cognitive, and social characteristics. Mixed graphical models including all variables were estimated using the R-package *mgm* discretely by sex. We also used the Walktrap cluster algorithm to identify differences in the network structure in terms of connectivity around frailty between the sex groups for further insights.

**Results:**

In both the networks for males and females, frailty correlated most strongly with nutritional status, presence of complex chronic disease, and self-efficacy, and exhibited proximity to decreased sleep quality and loneliness. However, frailty showed an association with suicidal ideation and the number of falls per year only in males, whereas it showed an association with functional disabilities only in females. The overall network connectivity around frailty was stronger with dense interactions (more edges) in the network for females than for males.

**Conclusions:**

The results signify the need for sex-group customized multi-domain assessments and interventions for the prevention and improvement of frailty among community-dwelling older adults.

## Background

Frailty is a geriatric syndrome characterized by progressive physiological decline of multiple organ systems, and the probability of its incidence increases with advancing age [1, 2]. With a rapidly increasing older adult population worldwide, the incidence of frailty is expected only to increase considerably. A recent systematic review and meta-analysis including > 120,000 older adults from 28 countries reported the estimated incidence of frailty to be 43.4 new cases per 1000 person-years [3].

Several studies have reported an association between frailty and adverse outcomes among older adults, including premature death, disability, falls, dementia, low quality of life, and nursing home admissions [4–7]. Considering these detrimental effects on health, frailty prevention and management are crucial. In particular, improved understanding of high-risk factors for frailty in this population is critical to informing interventions to prevent frailty and minimize its consequences.

Although several studies on factors associated with frailty exist, studying such associations across different populations is critical, particularly among sex-based groups, to inform tailored care considering specific sociocultural and health issues associated with each sex [8]. Moreover, the selection of the method is essential for the successful prediction of frailty, given frailty is affected by simultaneous interactions between multiple etiologic and modifying factors, an indication of its complex nature [9].

Network analysis is an approach that allows exploring simultaneous connections among several aspects associated with one or more health-related conditions [10]. This model is similar to a network, visualized as a set of associations between variables. Specifically, network analysis uses a graphical statistical method to understand multifactorial phenomena, allowing the distance and strength of correlations between different factors to be analyzed easily, which makes it superior to traditional analytic models [11]. In recent decades, particularly in the field of geriatrics, network analysis has emerged as a key technique for advanced research on various topics, including biological mechanisms involved in the aging process, validation of instruments, and various geriatric syndromes including frailty [9]. So far, 3 studies have used network analysis to investigate the characteristics of frailty and identified sex-specific correlates of this syndrome [8, 12, 13]. However, it remains underutilized despite its enormous potential.

South Korea (hereafter “Korea”) is one of the most rapidly aging countries. In addition, with changes in traditional values toward family, the proportion of older adults living alone is markedly increasing gradually in Korea [14]. According to a 2019 report by Statistics Korea, 34.2% of older adults aged ≥ 65 years live alone [15]. Aging and isolated living conditions have increased the risk of frailty among Korean older adults, which has received considerable attention in geriatric research. Yet, to the best of our knowledge, no studies from Korea have used network analysis to explore the aspects associated with frailty among older adults.

## Study aims

Accordingly, we aimed to characterize the sex-specific factors of frailty among Korean older adults using network analysis. Of note, our study targeted a sample of older adults living alone, who form a unique group of high-risk individuals. These individuals are the most vulnerable with regard to health status and present with a higher percentage of safety incidents and poor nutritional status, which explains the significant interest in frailty research [16].

## Methods

### Study participants

This study is based on a secondary analysis of cross-sectional data on community-dwelling older adults living alone in Siheung City in Korea. All participants met the following eligibility criteria: (a) age ≥ 65 years, (b) living alone in Siheung City, (c) capable of communicating orally and providing written informed consent, and (d) having the ability to understand the purpose and procedures of the study and having the willingness to participate.

Participants were drawn from the second-year cohort (cohort 2019, *N* = 1,041) of the original project which aimed to build community-based integrated services for older individuals living alone [17]. After excluding survey responses from 4 individuals with missing data, 1,037 individuals were evaluated. The original data were obtained via face-to-face interviews from August 12 to 23, 2019 at community welfare or health centers in Siheung City; the interviews used a validated survey questionnaire developed for the present study by research assistants. All research assistants were trained by the principal investigator in interview administration, study methodology, research tools, and individual assessment procedures. The corresponding author received coded data for secondary data analysis purposes from the principal investigator of the original project. Further details on the original project and the data collection process can be found elsewhere [17, 18].

### Measurements

#### Frailty

Frailty was assessed using the Korean Frailty Index (KFI), a comprehensive and multi-dimensional, community-validated frailty scale from the Korean Geriatrics Society [19]. The scale comprises 8 domains, each scored as 0 or 1: hospital admission, self-assessed general health, polypharmacy, loss of weight, depressed mood, incontinence, visual/auditory problems, and timed up-and-go test. The total score ranged from 0 to 8, with higher scores indicating higher frailty risk. Cronbach’s α for the developed KFI is 0.65. In the present study, the scale's reliability measured by Cronbach's α was 0.60. Evidence of its validity was provided by Jung et al. [20], who showed that the KFI is valid as a frailty assessment instrument among community-dwelling older Korean adults in regards to content, construct, and criterion validity when compared to the extensively researched Cardiovascular Health Study frailty scale.

#### Hypothesized risk factors of frailty: sociodemographic, health-related, mental and cognitive, and social factors

Sociodemographic factors included the following: a surviving child, education level, actual monthly cost of living, and social activity. Social activity signified the quality of social relationships and was assessed by inquiring whether the individuals visited certain types of community or religious centers.

Health-related factors included the presence of complex chronic disease, self-efficacy, functional disabilities, number of falls per year, nutritional status, alcohol consumption, smoking, and physical activity. We defined complex chronic disease as concurrent multiple health conditions arising from any of the following chronic diseases: hypertension, diabetes mellitus, hyperlipidemia, stroke, angina pectoris, rheumatoid arthritis, hepatitis, liver cirrhosis, asthma, chronic respiratory disease, chronic kidney disease, thyroid dysfunction, and chronic skin disease.

##### Self-efficacy

The 6-item Chronic Disease Self-Efficacy Scale (CDSE-6) [21] was used for self-efficacy assessment. The first 4 items are associated with confidence in preventing fatigue, pain or physical discomfort, emotional distress, and other symptoms or health concerns that interfere with the performance of desired activities. The remaining 2 items are associated with confidence in engaging in tasks other than medication intake for health management and for minimizing the effects of the disease on daily life. The item uses a 10-point scale (0: not at all confident; 10: totally confident); the scores of each item are added to obtain the total score. Cronbach’s α for the developed CDSE-6 is 0.91. Cronbach's α for the translated scale in the present study was 0.91. The CDSE-6 has been shown to have good construct validity with other self-efficacy measures including the 6-item and 17-item forms of the University of Washington Self-Efficacy Scale (*r* = 0.83 and 0.81, respectively), and is widely used in both clinical and research settings [22].

##### Functional disabilities

Functional disabilities were measured using the Korean Instrumental Activities of Daily Living (K-IADL) assessment tool, a well-developed and validated tool for easily assessing ADL among older adults [23]. K-IADL consists of 10 items each with a 3-point scale. The total score is computed by adding individual scores and dividing them by the number of questions, with higher scores representing poor performance. When developed, Cronbach's α for the scale was 0.94 and 0.87 in this study. Evidence of construct validity was provided by demonstrating the significant association between the K-IADL score and brain-disability grade (*r* = -0.68) by the original developers of the scale [23].

##### Nutritional status

Nutritional status was assessed according to the Mini Nutritional Assessment questionnaire-Short Form (MNA-SF) [24]. This short version eliminated time-consuming and subjective items from the full MNA and selected the following 6 items based on item correlation with the full MNA score, and with clinical nutritional status, internal consistency, completeness, and ease of administration [25]: food intake, involuntary loss of weight, mobility limitations, recent psychological stress or acute disease, neuropsychological issues (i.e., dementia and depression), and body mass index. The score ranges between 0 and 14; higher values signify better nutritional status. The MNS-SF demonstrates high internal consistency measured by Chronbach’s α (0.83) [24]. The scale's Cronbach's α was also satisfactory in the current study (0.81). Furthermore, the MNA-SF was found to have a high diagnostic accuracy relative to clinical nutritional status and is as good as the full MNA in predicting serum albumin [24]. Although the full MNA has shown sufficient internal consistency (Chronbach’s α, 0.71–0.83) in various older populations [26, 27], one systematic review addresses that MNS-SF has been the most appropriate nutrition screening tool for use among community-dwelling older adults [28].

##### Physical activity

Physical activity levels were evaluated using the International Physical Activity Questionnaire-Short Form (IPAQ-SF) [29]. IPAQ-SF assessed participant engagement in vigorous or moderate activities or walking in the past 7 days and the activity duration (hours and minutes). Each activity type was assigned a metabolic equivalent of task (MET) score: vigorous activities, 8.0; moderate activities, 4.0; and walking, 3.0. These values are added to calculate an individual’s overall MET score for a week.

Mental and cognitive factors included loneliness, suicidal ideation, decreased sleep quality, and cognitive function. Suicidal ideation (daily mean rating) and decreased sleep quality were assessed using a visual analog scale (VAS; 0–10 points), with 10 indicating the most severe symptoms.

##### Loneliness

Loneliness was measured with the Revised University of California Los Angeles Loneliness Scale (R-UCLA) [30], which comprises 20 items with 4-point scales. The revised scale is considered reliable across various populations with Cronbach’s α ranging from 0.89 to 0.94 [30] and 0.78 [31]. The current study used the Korean version translated by Kim and Kim [32]. The scale yields a maximum score of 80, with higher scores indicating more severe loneliness. The Korean version had satisfactory levels of test–retest reliability, internal consistency, and validity: the test–retest reliability computed in the study by Kim and Kim [32] was between 0.67 and 0.75, and the internal consistency measured by Cronbach’s α was 0.86. Our sample's R-UCLA's Cronbach's α was 0.91.

##### Cognitive function

The Mini-Mental State Examination-2 Standard Version (MMSE-2SV) scale was used to assess cognitive function [33]. The MMSE-2SV measures seven domains including memory registration, memory recall, orientation in time, orientation in place, attention and calculation, language, and drawing. A total score can range from 0 to 30; higher scores indicate better cognitive function. Raw scores of 0–17, 18–23, and 24–30 were used to categorize no, mild, and severe cognitive impairment, respectively (by using an algorithm that is commonly used by practitioners) [33]. The MMSE-2 demonstrates a sufficient internal consistency (Cronbach’s α, 0.66–0.79) [33] and has been validated in various studies [34–36]. The scale's Cronbach's α in our study was 0.71.

The social factor evaluated was perceived social support, which was measured using the Enhancing Recovery in Coronary Heart Disease Social Support Instrument (ESSI) [37]. The ESSI has been shown to have sufficient reliability (Cronbach’s α = 0.86) and has good construct validity with other social support measures including the Perceived Social Support Scale (*r* = 0.62) [37]. This study used the Korean version translated by Jeon et al. [38]. The example items include: ‘‘Do you have someone available who shows you affection and love?’’ and ‘‘Can you rely on anyone to offer you emotional support?’’ Each question answered as “yes” was scored 1, and all items are added to obtain a total score. A higher total score represents better social support. Cronbach's α for the translated scale was 0.85; in the present study, it was 0.76. The content validity of the translated scale was supported by a rigorous instrument development process that involved reviews and consensus from nursing, medical, and epidemiology experts [38].

### Statistical analysis

In the current study, the comprehensive statistical approach was undertaken to characterize the sex-specific factors that were associated with frailty. First, we assessed the simultaneous interactions between different frailty-associated factors and their mode of participation in the network model, discretely for males and females, with graphical visualization via network estimation. Moreover, a network cluster algorithm was applied to the network that was estimated in the previous step to investigate intergroup differences in connectivity between the network structures around frailty. Descriptive analyses were initially conducted to determine sample characteristics. All analyses were separately performed for each sex using the statistical programming language R and its available packages.

#### Network estimation

All variables (i.e., frailty and hypothesized risk factors for frailty) were included in a mixed graphical model implemented using the *mgm* package in R [39], which can accommodate binary, ordinal, and continuous variables. This model consists of nodes that symbolize each variable and edges between the nodes, which can be interpreted as conditional (partial) correlations, with values ranging from − 1 to + 1. A statistical penalty, namely the Least Absolute Shrinkage and Selection Operator (LASSO) [40], was applied to the *mgm* estimation procedure for the network to retain only the robust edges. The magnitude of the penalty is specified by the parameter “lambda,” which was selected with the Extended Bayesian Information Criterion (EBIC). EBIC uses a tuning parameter “gamma” (γ; lower γ = less conservative models), which was set to 0.25 by default to ensure a more conservative network estimation [41]. The *k* parameter was set to 2 to indicate pairwise relationships.

In addition to the network structure, we estimated the network's predictability using the “predict” function in *mgm* [42]. Predictability represents the shared variance of each node with all its direct neighbors using either the proportion of explained variance (R^2^) for continuous variables or normalized accuracy (nCC) for binary variables. Both metrics range from 0 to 1, with 1 implying all variance being explained. Networks were visualized using the *qgraph* package that used an average layout calculated by the Fruchterman-Reingold algorithm [43]. Green and red edges represent positive and negative LASSO-regularized partial correlations, respectively. Grey edges represent pairwise interactions wherein no sign is specified (i.e., interactions including categorical variables). Thicker or thinner lines indicate strong or weak correlations, respectively. The filled portions of the ring around each node indicate predictability.

We conducted a bootstrap analysis of network edge stability using the “resample” function in *mgm* (number of bootstrap samples = 100). The resulting sample distribution of all edges was plotted using the “plotRes” function in *mgm*. The plots exhibit the number of times an edge was estimated to be non-zero during the resampling process, along with the 5.0% and 95.0% quantiles of the estimates. The stability estimate, i.e., the percentage of bootstraps for which the edge was estimated to be non-zero, for the reported edge weights was also indicated.

#### Network clusters

We used the Walktrap cluster algorithm implemented in the *igraph* package to identify the subnetworks of strongly connected nodes around the nodes of interest (i.e., frailty) in the larger networks for males and females. The Walktrap cluster algorithm takes short “random walks” from the nodes of the graph, following the edges and their directions, and identifies clusters based on the number of times such a path remains inside the same group of nodes [44]. This result can be regarded as “communities” (or subnetworks), which are likely to circulate information among themselves with some level of separation from the rest of the network. The modularity ratio (known as Q-index) is used to evaluate the goodness-of-fit of the communities. Conventionally, modularity values between 0.3 and 0.7 (higher value = greater likelihood of non-random communities) indicate the presence of sub-clusters in the network [45].

## Results

**Fig. 1:**
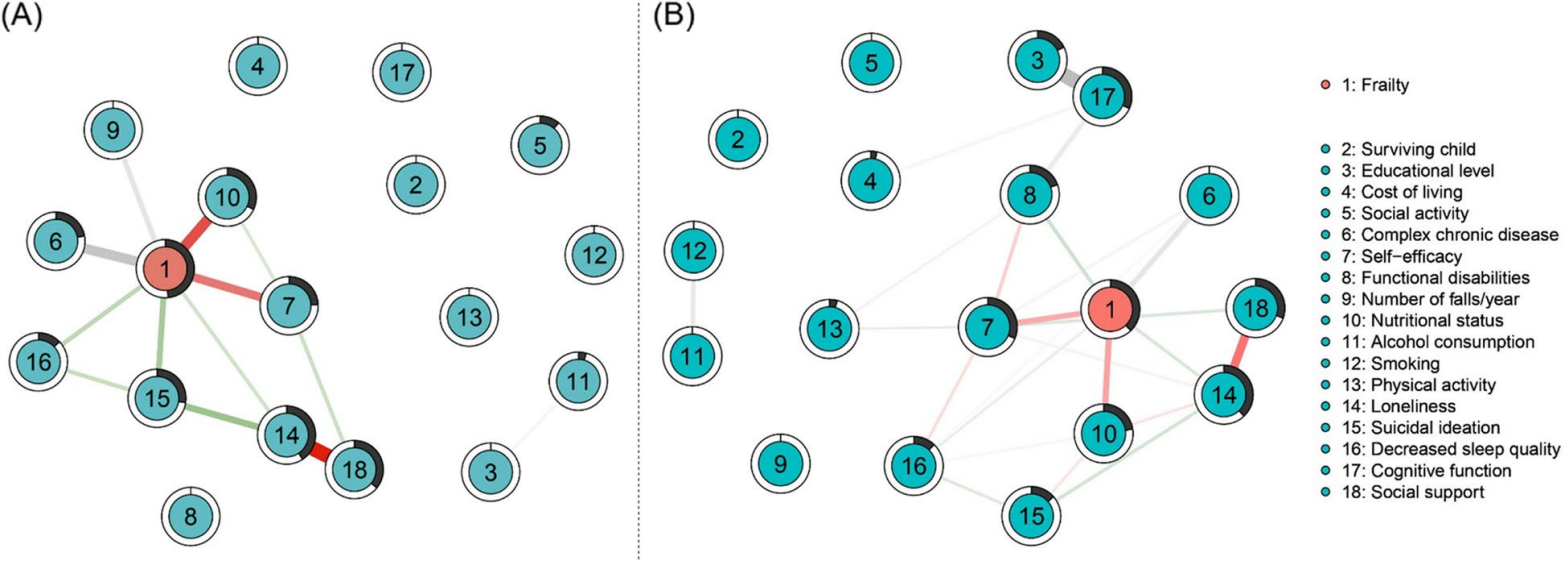
Mixed graphical model networks of the factors associated with frailty in older males (**A**) and older females (**B**) *Note.* Green and red edges represent positive and negative LASSO-regularized partial correlations, respectively. Grey edges represent pairwise interactions wherein no sign is specified (i.e., interactions including categorical variables). Thicker or thinner lines indicate strong or weak correlations, respectively. The filled portions of the ring around each node indicate predictability

Table 1 shows the background characteristics of the participants by sex. The mean ages of the males and females were 76.7 years (standard deviation [SD] = 5.66) and 79.7 years (SD = 5.29), respectively. The mean frailty scores of the males and females were 2.1 (SD = 1.65) and 2.9 (SD = 1.89), respectively. Table 2 summarizes the edge weights, stability of nonzero edges, and frailty predictability for males and females each. In the network for males, considering all the variables used in the network model, frailty most strongly correlated with nutritional status (edge weight = -0.30), presence of complex chronic disease (edge weight = 0.27), and self-efficacy (edge weight = -0.23). Stability analyses indicated robust associations between these edges (nonzero in 100.0%, 95.0%, and 100.0% of bootstrapped analyses, respectively). Frailty also was proximal to suicidal ideation (edge weight = 0.15), the number of falls per year (edge weight = 0.12), decreased sleep quality (edge weight = 0.12), and loneliness (edge weight = 0.08), with lesser edge weights and stability. These edges were nonzero in 82.0%, 73.0%, 47.0%, and 42.0% of bootstrapped models, respectively. Frailty predictability was 49.0%, as indicated by the black pie chart around the node representing frailty in (Fig. 1).

**Table 1:** Sex differences in general characteristics of older adults living alone

Variable	n (%) or mean ± standard deviation	
	Older males (*n* = 232)	Older females (*n* = 805)
Age (years)	76.7 ± 5.66	79.7 ± 5.29
Surviving child		
Yes	209 (90.1)	741 (92.0)
No	23 (9.9)	64 (8.0)
Educational level		
No education	29 (12.5)	385 (47.8)
Elementary school	53 (22.8)	263 (32.7)
Junior high school	51 (22.0)	93 (11.6)
High school and above	99 (42.7)	64 (7.9)
Actual monthly cost of living (KRW)	691,206.9 ± 493,205.96	517,199.0 ± 267,533.08
Social activity		
Yes	119 (51.3)	639 (79.4)
No	113 (48.7)	166 (20.6)
Presence of complex chronic disease		
Yes	129 (55.6)	596 (74.0)
No	103 (44.4)	209 (26.0)
Self-efficacy (CDSE-6)	39.2 ± 15.52	34.8 ± 15.49
Functional disabilities (K-IADL)	1.0 ± 0.11	1.1 ± 0.23
Number of falls per year		
No falls	190 (81.9)	569 (70.7)
One fall	24 (10.3)	137 (17.0)
Two or more falls	18 (7.8%)	99 (12.3)
Nutritional status (MNA-SF)	12.0 ± 2.12	11.8 ± 2.17
Alcohol consumption		
Never	66 (22.4)	615 (76.4)
Ex-drinker	58 (25.0)	92 (11.4)
Current drinker	108 (46.6)	98 (12.2)
Smoking		
Never	52 (22.4)	760 (94.4)
Ex-smoker	110 (47.4)	26 (3.2)
Current smoker	70 (30.2)	19 (2.4)
Physical activity (IPAQ-SF)	2092.8 ± 2651.94	1553.7 ± 2330.36
Loneliness (R-UCLA)	45.3 ± 13.69	41.3 ± 13.27
Suicidal ideation (VAS)	1.5 ± 2.76	1.1 ± 2.47
Decreased sleep quality (VAS)	2.7 ± 3.33	3.1 ± 3.39
Cognitive function (MMSE-2SV)		
No cognitive impairment	27.3 ± 1.75	26.6 ± 1.76
Mild cognitive impairment	21.2 ± 1.45	20.9 ± 1.75
Severe cognitive impairment	15.0 ± 2.00	14.0 ± 2.88
Social support (ESSI)	3.1 ± 2.05	3.9 ± 1.88
Frailty (KFI)	2.1 ± 1.65	2.9 ± 1.89

*CDSE* Chronic Disease Self-Efficacy Scale; *ESSI* Enhancing Recovery in Coronary Heart Disease Social Support Instrument, *IPAQ-SF* International Physical Activity Questionnaire-Short Form, *KFI* Korean Frailty Index, *K-IADL* Korean Instrumental Activities of Daily Living, *KRW* Korean Won, *MMSE-2SV* Mini-Mental State Examination-2 Standard Version, *MNA-SF* Mini Nutritional Assessment questionnaire-Short Form, *R-UCLA* Revised University of California Los Angeles Loneliness Scale, *VAS* Visual Analogue Scale

**Table 2:** Frailty predictability and frailty-risk factor edge weights/stability

	Older males (*n* = 232)	Older females (*n* = 805)
Overall frailty predictability	0.49	0.38
	Frailty-risk factor edge weights/stability	
Surviving child	X	X
Educational level	X	X
Actual monthly cost of living	X	X
Social activity	X	X
Presence of complex chronic disease	0.27 (95.0%)	0.21 (100.0%)
Self-efficacy	-0.23 (100.0%)	-0.24 (100.0%)
Functional disabilities	X	0.14 (74.0%)
Number of falls per year	0.12 (73.0%)	X
Nutritional status	-0.30 (100.0%)	-0.27 (100.0%)
Alcohol consumption	X	X
Smoking	X	X
Physical activity	X	X
Loneliness	0.08 (42.0%)	0.11 (100.0%)
Suicidal ideation	0.15 (82.0%)	X
Decreased sleep quality	0.12 (47.0%)	0.07 (76.0%)
Cognitive function	X	X
Social support	X	X

X = no edge between frailty and risk factors, % of 100 bootstraps for which the edge weight was non-zero in parentheses

Similar to the network for males, in the network for females, frailty showed a strong association with nutritional status (edge weight = -0.27), self-efficacy (edge weight = -0.24), and the presence of complex chronic disease (edge weight = 0.21; nonzero in 100.0%, 100.0%, and 100.0% of bootstraps, respectively). Frailty also showed relatively stable associations with loneliness (edge weight = 0.11) and decreased sleep quality (edge weight = 0.07). Of interest, in the network for females, an edge was found between frailty and functional disabilities (edge weight = 0.14), which was not observed in the network for males. The frailty-loneliness edge, frailty-decreased sleep quality edge, and frailty-functional disabilities edge were nonzero in 100.0%, 76.0%, and 74.0% of bootstraps, respectively. However, unlike the model only including males, no edges between frailty and suicidal ideation and between frailty and the number of falls per year were found in the model including females. The frailty predictability was 0.38.

**Fig. 2:**
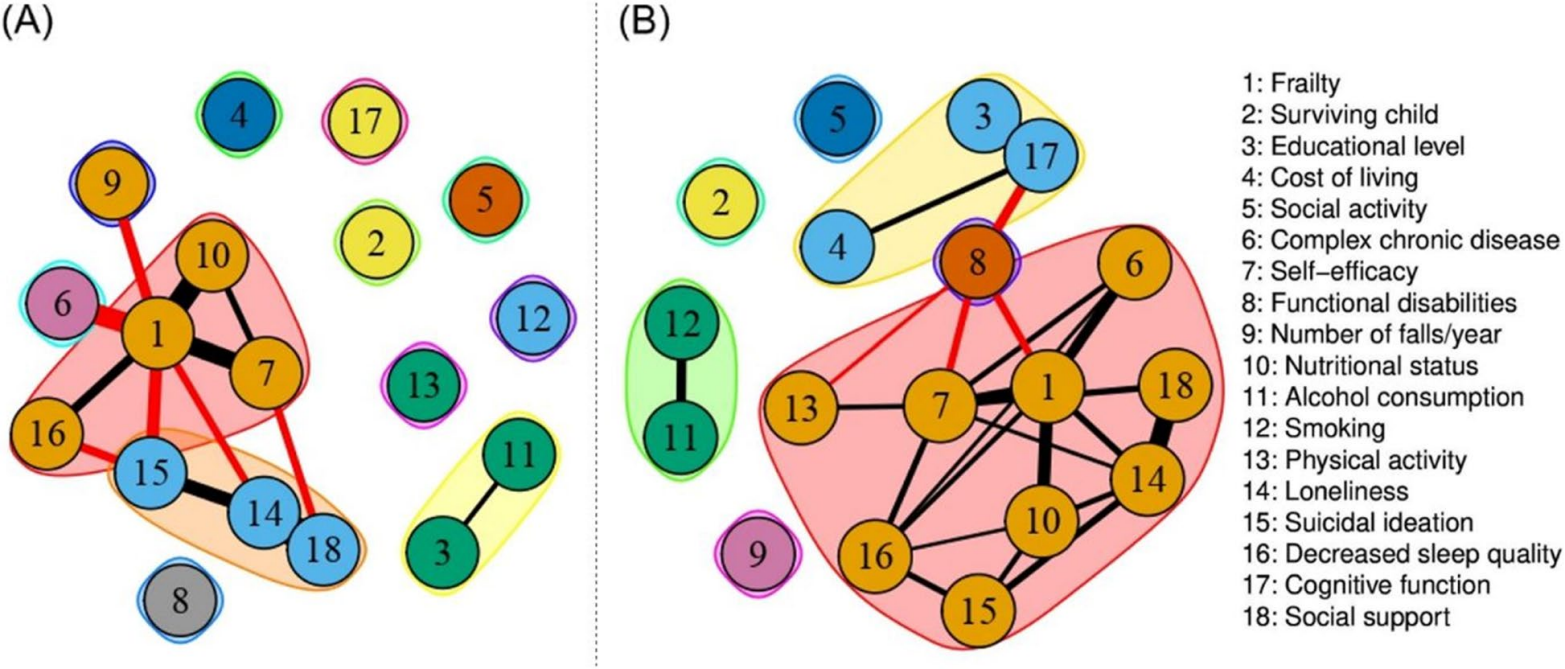
Network clusters for older males (**A**) and older females (**B**)

Figure 2 shows the clusters in the estimated networks. The clusters and their respective nodes are depicted in different colors. In the network for males, 3 clusters of strongly associated nodes were identified (modularity value = 0.31). In particular, frailty was located in the red cluster with self-efficacy, the number of falls per year, nutritional status, and decreased sleep quality. As shown in Fig. 2, the number of connections was greater between variables and frailty in females (modularity value = 0.40). In the network for females, frailty was also located in the red cluster that included the presence of complex chronic disease, self-efficacy, nutritional status, physical activity, loneliness, suicidal ideation, decreased sleep quality, and social support.

## Discussion

This study was used to characterize sex-specific frailty-associated factors among Korean older adults living alone based on network analysis. First, the dynamic interactions between different sex-specific factors associated with frailty were identified discretely via network estimation, and these were subsequently used in the Walktrap cluster algorithm to illuminate the sex-specific differences in the network structure in terms of their connectivity to frailty.

In both the networks for males and females, frailty correlated most strongly with nutritional status. This is an important finding as the risk of malnutrition is notably prevalent in older individuals living alone [46]. Evidence suggests that malnutrition increases the age-related loss of muscle mass and strength, which can contribute to the development of sarcopenia and subsequent physical impairment, both of which represent critical components of the frailty syndrome [47]. Indeed, nutrition support has been consistently targeted in interventions to slow or reverse pre-frailty in community-dwelling older adults [48]. Of note, Bollwein et al. [47] recommended profoundly describing the interdependency of these two concepts for more successful strategies; for instance, investigating the relationship between frailty and the MNA in subscores, categories, or single items, which could also be addressed using network analyses.

The link between frailty and self-efficacy found in both the networks for males and females also merits closer attention, as it has been increasingly recognized in the literature [49–51]. For instance, Doba et al. [49] reported a significant cross-sectional association between frailty and self-efficacy among Japanese older adults, recommending that a self-efficacy assessment could be useful for successful aging in older adults. In addition, in a 12-month pilot randomized controlled trial including 117 Taiwanese older adults [50], it was reported that problem-solving therapy based on self-efficacy led to a short-term 44.0% improvement in frailty status.

Furthermore, the proximity of frailty to loneliness in both of the network models is in line with the previous evidence, which implicates loneliness as a crucial factor in the onset and progression of frailty. One of the most discussed mechanisms that underlies this association is low gait speed and mobility as well as increased difficulty in performing activities of daily living, all of which are associated with loneliness and predispose individuals to sarcopenia—an age-related reduction in muscle mass, strength, and function that constitutes a major trigger of frailty [52–54]. At the biological level, frailty and loneliness share a proinflammatory phenotype [55]. Furthermore, high frailty led to increased loneliness [56]. As their interaction was found to be strong and direct, researchers argued that psychological factors must be considered inherent to frailty, and even average levels of loneliness should not be regarded as acceptable, and instead, should be actively addressed [57]. This is particularly relevant for older adults living alone, where the prevalence of loneliness is higher than in general cohorts.

Frailty also exhibited a close relation to sleep quality for both sexes. Some argue that decreased sleep quality is a marker of various comorbid conditions such as functional loss, depression, and cardiovascular diseases—which by themselves are well-known risk factors of frailty—and this can partially explain the observed association [58, 59]. Others argue that decreased sleep quality relates to renal dysfunction, which may induce protein degradation and muscle proteolysis, thereby increasing frailty risk [60]. Yet, the relationship between frailty and sleep quality has been understudied; one systematic review has found limited research in this field [61]. Owing to the indeterminate role of sleep in frailty pathophysiology, the majority of nurse-led care programs for frail older individuals barely incorporate sleep-related screening and interventions [62–64], which calls for further evidence.

Interestingly, in the network analysis, frailty showed a direct association with suicidal ideation only in males. This corroborates accumulated research demonstrating the significant association between performance on frailty measures (e.g., muscle weakness, gait speed) and the levels of suicidal ideation found in males [65, 66]. Indeed, frailty characteristics such as fatigability, muscle mass loss, or decreased physical functions in older adults can lead to withdrawal from social relationships and resulting isolation may promote suicidal thoughts [67]. Despite previous publications, sex discrepancy in the link between frailty and suicidality remains under-researched. One possibility for this discrepancy is that older females are known to use more effective social coping strategies, which may act as a buffer against potential suicidality [68]. Korean studies consistently argued that older females have fewer negative experiences after divorce or bereavement than males [69], or that older females living alone are better at developing bonds with family and friends than older males living alone [70]. In fact, our network analysis findings on frailty being clustered with social support only for females indirectly supports this argument.

The number of falls per year was also associated with frailty only in males, which is consistent with the previous network investigation [9]. This may be due to the vulnerabilities that are specific to males. For instance, it has been argued that low testosterone levels in older males are related to a loss of muscle mass and function and that these age-related changes might be associated with falls [71]. Yet, our findings do not imply that males fall more, but instead, the prevention of falls may be of greater importance in frailty intervention for older males than for older females.

Contrastingly, frailty was associated with functional disabilities only in females. Our results align with the study by Puts et al. [72] which showed that worsening frailty influenced functional decline in females, but not in males. Our results can be discussed from multiple perspectives. In cultural contexts, it is common for the current generation of older Korean females to have devoted their lives to household care, their time mainly being spent in restricted private spaces, and thus they usually initially present with worse functional conditions [73]. Moreover, higher frailty levels in older females are explained by their generally higher levels of comorbidities, including osteoarticular diseases that affect mobility [74, 75]. Therefore, it may be plausible to find the closer, overlapping association between frailty and functional loss more easily in the older female group.

According to the Walktrap cluster algorithm, the overall network connectivity around frailty was stronger with dense interactions in the network for females than for males. In the network for males, frailty clustered with decreased sleep quality, self-efficacy, number of falls per year, and nutritional status. In the network for females, frailty clustered with social support, loneliness, suicidal ideation, decreased sleep quality, self-efficacy, presence of complex chronic disease, nutritional status, and physical activity. The abovementioned findings of this study indicate the following important implications.

First, the findings could partly explain the increased vulnerability of females to develop frailty. From a network perspective, a denser network implies that a person is more “trapped” in a disordered state compared to someone with a less dense network [76]. In our study context, more densely connected networks may feature stronger feedback among the traits/symptoms, and thus might be associated with greater vulnerability to frailty. In fact, literature over the decades has documented clear sex differences in frailty, with females almost always having higher frailty prevalence and incidence [3]. Second, the findings may indicate that nodes identified in the clusters around frailty for each sex group can potentially constitute useful intervention points that should be prioritized. Network studies suggest that stronger connections between traits/symptoms in a network can be expected to lead to more changes (owing to traits/symptoms affecting each other) [77]. In particular, a cluster may pinpoint a group of nodes that can be easily influenced when a node included in the cluster changes state [77]. For instance, changes in self-efficacy (the most connected node with high predictability in the female cluster) can result in changes in frailty level (and vice versa) more quickly than others (i.e., assuming that this relationship is bidirectional), making it a fruitful point for intervention. Third, the findings support the efficacy of a multi-domain, multifaceted intervention for addressing the frailty issue, as compared to that of a single-domain or a direct intervention for frailty. A recent systematic review revealed that an intervention that combined muscle strength training and protein supplementation was most effective in delaying or reversing frailty and was the easiest intervention to implement in primary care [78]. In the study by Ng et al. [79], a multi-domain approach that combined nutritional, physical, and cognitive interventions significantly reduced progression to or worsening of frailty in pre-frail and frail older adults. Notably in this study, stronger connectivity around frailty with more edges in the female’s network demonstrates that more multifaceted approaches are warranted to address frailty in this population.

Finally, it may be valuable to further discuss the stronger network connectivity around frailty that is observed in females. A rationale for this may be the possible existence of cognitive frailty, which is defined as the simultaneous presence of both physical frailty and cognitive impairment without dementia [80]. In fact, besides the higher frailty in females than in males, many females in this study showed mild cognitive impairment (MMSE-2SV score, 23.6 ± 4.59) whereas males had scores in the normal cognitive range (26.2 ± 3.00). Cognitive frailty is fairly a new construct, is known to be more prevalent in females and, compared to frailty or cognitive impairment used separately, has been shown to be a better predictor of adverse health outcomes, increased disability, and mortality among older people [81, 82]. Highly heterogeneous risk factors underlie this construct for which multi-domain interventions are particularly beneficial [80], which may partially explain our findings in females. As yet, the expansion of this discussion warrants a clear operationalization of cognitive frailty, which is beyond the scope of our paper.

### Limitations and implications

There are some limitations to our study. First, the generalizability of our findings is limited to populations similar to those chosen for this study. Second, the directionality of the edges cannot be assessed due to the cross-sectional nature of the data. A longitudinal study, by contrast, can provide answers to the true meaning of the proposed interactions. Second, compared to the network for females, the network for males included a smaller number of participants, and this may result in a network structure with inferior performance in tests to evaluate the accuracy of the inferences. Thus, caution should be exerted when interpreting these results. Third, most of the variables employed in this study were obtained by self-report, and the interactions could be affected by how the questions were developed. Fourth, we used the frailty index rather than the frailty phenotype. The former offers a view consisting of an accumulation of deficits (taking into account signs/symptoms, diseases, and disabilities as deficits), while the latter represents the frailty concept with regard to biologically determined patterns [12]. Possibly, the strength of the associations between risk factors might have changed if the frailty phenotype had been used, which might be more carefully considered in future research. Lastly, this study could not compare the two estimated networks formally with network comparison statistics such as the network comparison test [83], as the network comparison test has not been validated for mixed data with ordinal, binary, and continuous variables. Recently, Haslbeck [84] introduced a new technique to explore group differences in network models based upon a moderation analysis using the R-package *mgm*—which can be utilized in the near future to substantiate our findings.

Despite the abovementioned limitations, our study yields clinical implications on two major points. First, the results suggest that frailty is associated with different risk factors for both sexes, which speaks to the importance of tailored assessment and intervention. Moreover, the findings based on a cluster algorithm emphasizes the need for multifaceted approaches to address frailty, especially for older females. Thus, frail older adults may benefit from management by a wider multi-disciplinary team. Indeed, a review of the literature suggests that, among the existing multifactorial interventions of frailty, the ones delivered by a multidisciplinary team consisting of geriatricians, physiotherapists, rehabilitation physicians, nurses, and dieticians improved frailty status and helped maintain physical function in frail older adults [85, 86].

## Conclusion

Using a sample of community-dwelling older adults living alone, we sought a more profound understanding of sex-specific correlates of frailty using network analysis. Overall, the findings support the multifactorial etiology of frailty for each sex group, which in turn informs the development of tailored multi-domain assessment and interventions. In the future, this network analytic approach to study frailty can be extended to other groups, including different socioeconomic strata, regional populations, and more vulnerable older adults.

## Data Availability

The datasets used and analyzed during the current study are available from the corresponding author on reasonable request.

## Abbreviations

CDSE: Chronic Disease Self-Efficacy Scale
EBIC: Extended Bayesian Information Criterion
ESSI: Enhancing Recovery in Coronary Heart Disease Social Support Instrument
IPAQ-SF: International Physical Activity Questionnaire-Short Form
KFI: Korean Frailty Index
K-IADL: Korean Instrumental Activities of Daily Living
KRW: Korean Won
LASSO: Least Absolute Shrinkage and Selection Operator
MET: Metabolic equivalent of task
MMSE-2SV: Mini-Mental State Examination-2 Standard Version
MNA-SF: Mini Nutritional Assessment questionnaire-Short Form
VAS: Visual Analogue Scale
R-UCLA: Revised University of California Los Angeles Loneliness Scale
